# *Sarcoptes scabiei*: genomics to proteomics to biology

**DOI:** 10.1186/s13071-016-1663-6

**Published:** 2016-07-01

**Authors:** Larry G. Arlian, Marjorie S. Morgan, S. Dean Rider

**Affiliations:** Department of Biological Sciences, Wright State University, 3640 Colonel Glenn Hwy, Dayton, OH 45435 USA

**Keywords:** *Sarcoptes scabiei*, Scabies mite, Genome sequence, Biological processes

## Abstract

**Background:**

The common scabies mite, *Sarcoptes scabiei* is a cosmopolitan parasite of humans and other mammals. An annotated genome of *Sarcoptes scabiei* var. *canis* has been deposited in the National Center for Biotechnology Information (NCBI) and VectorBase and a proteomic analysis of proteins in extracts of mite bodies and eggs from this strain has been reported. Here we mined the data to identify predicted proteins that are known to be involved in specific biological processes in other animals.

**Results:**

We identified predicted proteins that are associated with immunomodulation of the host defense system, and biological processes of the mite including oxygen procurement and aerobic respiration, oxidative metabolism, sensory reception and locating a host, neuronal transmission, stressors (heat shock proteins), molting, movement, nutrient procurement and digestion, and excretion and water balance. We used these data to speculate that certain biological processes may occur in scabies mites.

**Conclusion:**

This analysis helps understand the biology of *Sarcoptes scabiei* var. *canis* and adds to the data already available in NCBI and VectorBase.

**Electronic supplementary material:**

The online version of this article (doi:10.1186/s13071-016-1663-6) contains supplementary material, which is available to authorized users.

## Background

The scabies mite, *Sarcoptes scabiei*, is a permanent obligate ectoparasite that lives and reproduces in the epidermis of skin of humans and many other mammalian hosts. Scabies is an important, neglected disease afflicting populations worldwide causing significant human and animal morbidity and mortality. Although there has been much progress in understanding the mite biology and interaction with its host, much is yet unknown.

A recent proteomic analysis of *S. scabiei* var. *canis* provided a listing of some of the proteins found in an aqueous extract of mite bodies, an extract of insoluble pellet of mite bodies, and an aqueous extract made from scabies mite eggs [1]. Likewise, the genome has been sequenced for this strain of *S. scabiei* [2]. The genomic and proteomic data are available at the National Center for Biotechnology Information (NCBI) and VectorBase. The genomic and proteomic data for specific aspects of the biology of this mite species provides a greater understanding of its physiology and the biochemical pathways associated with specific functions. Also, it may provide the basis for further research of this parasite. In the present research, we focused on questions about the biology of scabies mites and then searched for proteins likely associated with these biological processes. With these data it is now possible to identify groups of predicted proteins and to postulate certain biological processes and mechanisms used by these mites to live on and interact with their hosts.

## Methods

We have previously reported the sequencing of DNA from the scabies mite, *Sarcoptes scabiei* var. *canis*, and the assembly of a draft genome for this organism [2]. All data for this project were deposited into the National Center for Biotechnology Information (NCBI) under BioProject PRJNA268368. Data are also available through VectorBase at www.vectorbase.org. Along with the draft genome, the sequences of 10,470 predicted *Sarcoptes scabiei* var. *canis* proteins were also deposited with accession numbers KPL93347 to KPM12096. The production of > 150 of these predicted proteins was previously confirmed by mass spectrometry [1] and an additional 50 proteins have since been identified using identical methods.

Most of the data tables presented in this manuscript were constructed using keyword searches of the NCBI *S. scabiei* protein database which includes the 10,470 *S. scabiei* var. *canis* sequences described above as well as ~270 additional sequences for proteins from scabies mites collected from other hosts (mostly humans and pigs). These searches returned hits when the keyword was part of the name assigned to the protein as well as when the keyword was found in the annotated record for a protein. For example, a search for “heme” returned both Heme peroxidase-like protein (where “heme” is in the name) and globin-like protein 1 (since the protein record indicates that a “heme-binding site” is part of the predicted structure). Proteins whose production was confirmed by mass spectrometry (MS) are noted in the supplementary tables (Additional file 1: Tables S1–S13).

BLAST2GO version 3.2 [3] was used to identify GO terms from interpro domains identified in all *S. scabiei* var. *canis* proteins that were not designated as “hypothetical” proteins in the proteome. These data are available in Additional file 2. GO terms grouped by biological process, cellular location, and molecular function were exported and used in keyword searches to help identify proteins of interest.

Molecular phylogenies generated for groups of related proteins utilized alignments generated by ClustalOmega (available at http://www.ebi.ac.uk/Tools/msa/clustalo/), followed by reconstructions of Neighbor Joining trees using ClustalW2 Phylogeny. Fig Tree v1.4.2 was used to render the phylogenetic tree images.

## Results and discussion

### Immunomodulation

Molecules in extracts made from scabies mites have been shown to modulate the secretion or expression of cytokines, chemokines and cell adhesion molecules by cells of the epidermis and dermis of the skin and circulating blood lymphocytes [4–15], and inhibit the complement pathway of the host [16]. These data suggested that some aspects of this modulation delay the innate and adaptive immune responses to these mites and are responsible for the lack of clinical signs in the host for 4–8 weeks before skin symptoms are visible and patients seek diagnosis and treatment. Specifically, human peripheral blood mononuclear cells (PBMCs) stimulated with a whole body extract of scabies mites were shown to produce interleukin-10 (IL-10) but not IL-2 and IL-4 [9]. This cytokine profile suggested that the stimulated cell population consisted of T-regulatory (Treg) cells and not T-helper-1 (Th-1), Th-2, and Th-17 phenotypes. Studies have shown that cystatin, a 14 kDa protein in extracts of the parasitic round worm, *Ascaris lumbricoides*, up-regulated expression of IL-10 and transforming growth factor-β (TGFβ) from splenocytes from mice [17]. The genome for scabies mites contains two genes (QR98_0079060 and QR98_0079070) coding for cystatin-like proteins (KPM09372 and KPM09373) so it can be predicted that scabies mites may produce cystatin-like proteins and these proteins may be responsible for the up-regulated secretion of IL-10 observed from the Treg cells stimulated with a scabies mite extract. IL-10 is anti-inflammatory and immune-suppressive and among other things, down-regulates a T-cell mediated immune response by depressing the expression of MHC II on the surface of antigen-presenting cells (APCs) and thus the interaction of the MHC II/antigen complex on the APCs with the T-cell receptor needed to stimulate T-helper cells.

Interestingly, several tick species including *Ixodes scapularis*, *Dermacentor variabilis*, *Rhipicephalus microplus*, *Haemaphysalis longicornis* and *Ornithodoros moubata* (soft tick) have predicted cystatin-like proteins with some homology (< 31 % identity) to the scabies mite molecules (DELTA-BLAST search of KPM09372 and KPM09373 *vs* non-redundant protein sequences restricted to Acari, 16 Mar 2016). The two predicted scabies mite cystatin-like proteins also show limited (31 %) identity to each other. The gene for one of the predicted scabies mite proteins has a secretion signal (QR98_0079060).

Likewise, macrophage migration inhibitory factor (MIF) genes have been identified in both ticks and scabies mites [2, 18]. MIF is a chemokine that regulates macrophage and other leukocyte migration [19]. A MIF gene from scabies mites has high nucleotide sequence homology with a MIF gene from the ticks *D. variabilis*, *Amyblomma americanum* and *H. longicornis* [18]. Although the gene nucleotide sequences differed slightly, the putative amino acid sequences for the scabies mite and the tick *D. variabilis* MIF proteins are identical.

Many genes for homologs of salivary gland proteins from ticks and other blood-sucking arthropods are present in the scabies mite genome [2]. These include ferritins, cathepsins, glutathione-S-transferases and thioredoxins. Genes for multiple tetraspanins that are involved in cell adhesion, cell migration and proliferation [20] are also present. Scabies mites have ten tetraspanin-like proteins and eight others with tetraspanin domains (Table 1). Scabies mites also have several genes that code for production of calmodulin-like proteins [2], which are calcium-binding proteins found in tick saliva [21]. The presence of many of these putative sialoproteins, including camodulins and calreticulin, in scabies mite body extracts was also verified by mass spectrometry [1].

**Table 1:** *Sarcoptes scabiei* var. *canis* proteins containing tetraspanin domains. Proteins were identified by NBCI protein database search of “*Sarcoptes scabiei* [and] tetraspanin” on 28 Mar 16

Name assigned to *S. scabiei* predicted protein	# aa	Accession #
Tetraspanin-like protein 1	271	KPM03168
Tetraspanin-like protein 2	209	KPM03650
Tetraspanin-like protein 3	201	KPM04223
Tetraspanin-like protein 4	240	KPM04224
Tetraspanin-like protein 5	228	KPM06000
Tetraspanin-like protein 6	382	KPM08766
Tetraspanin-like protein 7	249	KPM08935
Tetraspanin-like protein 8	88	KPM11003
Tetraspanin-5-like protein	338	KPM02620
Tetraspanin-11-like protein	248	KPM04544
CD151 antigen-like protein	148	KPM06528
CD63 antigen-like protein	236	KPM04484
CD81 antigen-like protein	288	KPM04961
Hypothetical protein QR98_0011670	152	KPM02748
Hypothetical protein QR98_0030280	252	KPM04578
Hypothetical protein QR98_0032560	272	KPM04802
Hypothetical protein QR98_0065550, partial	302	KPM08042
Hypothetical protein QR98_0076100	176	KPM09080

### Oxygen requirements

Scabies mites represent an unusual taxon of arthropods and metazoan animals that require oxygen (aerobic) but lack an organized respiratory system for obtaining it. They belong to the Astigmata group of Acari since no specialized surface or organ involved in oxygen uptake has yet been described or proposed. Scabies mites do not seem to have homologs for genes related to the known development of trachea that have been identified in *Drosophila* [22]. Interestingly, the scabies mite genome contains a homolog for the gene “scribbled” which in *Drosophila* promotes the development of both tracheae and salivary glands [22]. Apparently, the downstream genes representing the tracheal arm of this pathway are absent in scabies mites while the salivary gland pathway genes are present. In contrast, despite lacking the trachea-promoting genes identified in *Drosophila,* ticks and some other mites have tracheae [23, 24] so in some Acari there may be an alternative pathway for developing tracheae.

The oxygen requirements for scabies mites are low and were reported to be 0.002 and 0.0008 μl O_2_/h for female and male mites, respectively [25]. In comparison, the closely related astigmatid house dust mite, *Dermatophagoides farinae*, is of similar size and utilizes 0.009 μl O_2_/h for females [26]. Neither the source nor the mechanism for obtaining oxygen is known for any of these astigmatid mites. In the case of house dust and storage mites that are terrestrial, the source of oxygen is most likely ambient air. It is not clear what the source of oxygen is for scabies mites while they are burrowing in the epidermis but presumably when they wander on the surface of the skin, they obtain oxygen from the ambient air. How much O_2_ is available in the burrow in the skin is unknown. If the mites ingest serum as they burrow, dissolved oxygen in this fluid could provide the oxygen they need along with nutrients. In humans, the partial pressure of oxygen in the arterial blood and serum in the surrounding tissues that bathes cells is about 100 mmHg (0.003 μl O_2_/μl fluid) and drops to 40 mmHg (0.0012 μl O_2_/μl fluid) in venule blood. Could this be enough to meet their needs? These mites are too small to perform many physiological studies that would provide clues to how they obtain oxygen. Histology (electron microscopy) of both scabies and house dust mites provides no clues. However, genomic and proteomic data and associated genes and protein homologs that are associated with the process of obtaining oxygen and eliminating carbon dioxide in other organisms may provide some hints for these processes in scabies mites.

Regardless of how oxygen enters the mite body, scabies mites possess a wide variety of proteins typically involved in oxygen transport and utilization in other animals. Our analysis has predicted many proteins that may have an oxygen binding/transporting function in *S. scabiei* (Additional file 1: Table S1). Among these are more than 150 predicted proteins that contain heme, an oxygen-binding prosthetic group. Four genes code for globin proteins (QR98_0021760 = KPM03742 = globin-like protein 1; QR98_0035350 = KPM05076 = globin-like protein 2; QR98_0086180 = KPM10070 = cytoglobin-1-like protein; and QR98_0042210 = KPM05052 = neuroglobin-like protein) that could be involved in the transport of oxygen. The predicted proteome also contains a complete set of oxidative respiration (tricarboxylic acid cycle) enzymes (several of which were previously identified by MS) [1] as well as > 130 enzymes involved in oxidative/reduction processes (Additional file 1: Table S2).

Thus, scabies mites require oxygen and they have many genes and predicted protein products associated with this requirement but the anatomy and biochemistry of getting O_2_ into the body and to the cells are unknown.

### Post-embryonic development/molting

The life-cycle of scabies mites includes the egg, larva, protonymph, tritonymph and adult male or female [27]. Larvae hatch from eggs, feed, grow, undergo metamorphosis and molt to protonymphs. The process is repeated for protonymphs that give rise to tritonymphs and tritomymphs that give rise to adults. The molting process is well detailed for insects where it is regulated by ecdysteroids, and presumably, it is similar in mites. It involves digestion and reabsorption of some of the old cuticle (procuticle and endocuticle if both are present) and development of a new cuticle (procuticle) and varied degrees of sclerotization to harden the new procuticle. Hardening involves cross-linking of linear proteins and chitin molecules, the major components of the cuticle [28, 29]. Chitin is a polymer of N-acetyl-D-glucosamine molecules that are linked by β-1,4 covalent bonds. Chitin can be hydrolyzed by chitinase and exochitinase (or β-N-acetylglucosaminidase) [30]. Chitin synthase promotes polymerization of the linear chitin molecule. Proteases hydrolyze the protein filaments. Many enzymes and biochemical pathways and several hormones are involved in the synthesis, translocation and degradation of the chitin and protein in the cuticle during the molting process. The scabies mite proteome contains 16 proteins that are predicted to serve as structural constituents of the cuticle (Table 2). This proteome also contains two chitin synthase-like proteins and four chitin deacetylase-like proteins (the latter catalyzes cleavage of acetate from the glucosamine molecule). In addition, there are 12 chitinase-like proteins, two (Sar s 15 and 18) that are homologs of house dust mite allergens (Additional file 1: Table S3). The scabies mite proteome also contains several glucosidases but it is unknown if they may play a role in degrading the cuticle. The genome also contains homologs of all of the enzymes known to be involved in ecdysteroidogenesis [31], indictating ecdysteroids are likely to regulate development in this mite (Table 3).

**Table 2:** Structural constituents of *Sarcoptes scabiei* var. *canis* cuticle. Proteins were identified by NBCI protein database search of “*Sarcoptes scabiei* [and] cuticle” on 11 Feb 16

Name assigned to *S. scabiei* predicted protein	# aa	Accession #
Cuticle protein-like protein 1	104	KPL98302
Cuticle protein-like protein 2	663	KPM03015
Cuticle protein-like protein 3	168	KPM06751
Cuticle protein-like protein 4	404	KPM08892
Cuticle protein-like protein 5	225	KPM10980
Cuticle protein-like protein 6	341	KPM10998
Cuticle protein-like protein 7	314	KPM11880
Cuticular protein-like protein 9	160	KPM09762
Cuticular protein-like protein 8	360	KPM02447
Cuticular protein-like protein 10	189	KPM09771
Cuticular protein-like protein 11, partial	245	KPM10754
Cuticle protein 57A-like protein 1	107	KPM08966
Cuticle protein 57A-like protein 2	189	KPM08973
Cuticle protein 92A-like protein	121	KPM08994
Cuticle protein viking-like protein	584	KPM06292
Structural constituent of cuticle-like protein	214	KPM09986

**Table 3:** *Drosophila melanogaster* genes involved in ecdysteroidogenesis and their predicted *Sarcoptes scabiei* var. *canis* gene and protein homologs. Adapted from [31]

*D. melanogaster* gene	*S. scabiei* gene homolog	Name assigned to *S. scabiei* predicted protein	Accession #
neverland	QR98_0055560	Rieske domain-containing protein	KPM07074
shroud	QR98_0023390	11-cis retinol dehydrogenase-like protein	KPM03900
spook/spookier	QR98_0048390	cytochrome P450-like protein 16	KPM06364
phantom	QR98_0078290	cytochrome P450-like protein 20	KPM09295
disembodied	QR98_0104600	cytochrome P450-like protein 26	KPM11882
shadow	QR98_0023200	cytochrome P450-like protein 10	KPM03882
shadow	QR98_0044620	cytochrome P450-like protein 15	KPM05989
shade	QR98_0022240	cytochrome P450-like protein 9	KPM03790

Compounds that target and interfere with specific steps in the formation of the cuticle and the molting process have been developed for use as insecticides to control some insect species. As more is learned about these processes in mites and in particular scabies mites, specific aspects of these processes may be targets for developing new methods for controlling these mites on their hosts.

### Muscle physiology

A general description of the mechanism for striated muscle activation and contraction can be found in many general physiology textbooks. Presumably scabies mites have striated muscles with morphology and contractile physiology similar to those of other arthropods and vertebrates. These would include actin and myosin protein fibers and the interaction sites involving dihydropyridine receptors, ryanodine receptors, calcium channels and pumps, tropomyosin, troponin subunits T, C, and I and calsequestrin. The fundamental mechanism based mostly on vertebrate studies, is that action potentials (depolarization), traveling into T-tubules mediated through dihydropyridine receptors and ryanodine receptors, cause opening of calcium channels in the sarcoplasmic reticulum (SR) membrane and the release of calcium from the SR into the myoplasm [32–34]. Activation of the actin and myosin contraction complex that results in sliding of the filaments to shorten the sarcomere (thus muscle), is calcium mediated and involves a calcium flux from the SR lumen into the myoplasm around the actin and myosin filaments. Relaxation of the muscle results from lower calcium concentration in the myoplasm by sequestration of calcium back into the SR. Lower calcium concentration in the myoplasm is accomplished by transmembrane proteins that pump calcium from the myoplasm back into the SR lumen where it binds to calsequestrin [35]. The presence of actin, myosin and tropomyosin in whole body extracts of scabies mites has already been confirmed by mass spectrometry [1]. The predicted proteome of scabies mites includes multiple actins (many of which may be involved in cytoskeletal movement) and myosins, tropomyosin, troponin T-, C- and I-like proteins, ryanodine receptors, and numerous calcium pump proteins (Additional file 1: Table S4). Also, the scabies mite predicted proteome includes the calcium-binding proteins, calmodulin and calsequestrin-2-like proteins, as well. These proteins play a key role in sequestering calcium in the SR and in muscle contraction [35]. All this taken together is indirect evidence that muscle contraction and movement in scabies mites are accomplished by anatomy and contractile mechanisms that are similar to those used by insects, other arthropods and vertebrates.

### Calcium-binding proteins

Approximately 143 calcium-binding proteins are predicted in the *S. scabiei* proteome and these are listed in Additional file 1: Table S5. Many such as calsequestrin and calmodulin, that are important in sequestration of calcium ions in the SR, are essential to muscle function and have already been mentioned. Another group appears to be low-density lipoprotein receptor-like proteins while many are hypothetical proteins of undetermined function.

### Heat shock proteins and others involved in protein folding and conformation

Heat shock proteins (HSPs) are a large family of intracellular proteins that have multiple functions including acting as chaperones and maintaining the conformation and folding/unfolding of proteins, assembly of protein complexes, and transporting and sorting of proteins [36–38]. Each protein is designated by its molecular weight in kilodaltons. They are ubiquitous in organisms and are generally expressed when organisms encounter stressful conditions. HSPs with molecular weights, including 22, 23, 26, 27, 40, 60, 68, 70, 75, 78, 83 and 90, have been reported in a variety of insect species [37]. We found a large number of heat shock proteins in the proteome of scabies mites (Table 4) and the production of many of them has been confirmed by MS analysis [1]. Eight heat shock protein 70-like proteins (Sar s 28) are homologs of house dust mite allergens. HSP 10, 20, 60 and 90 family members are also present in the scabies mite proteome. This proteome also contains many other chaperone-type proteins (Additional file 1: Table S6).

**Table 4:** *Sarcoptes scabiei* var. *canis* heat shock proteins. Proteins were identified by NBCI protein database search of “*Sarcoptes scabiei* [and] heat shock” on 11 Feb 16

Name assigned to *S. scabiei* predicted protein	# aa	Accession #
10 kDa Heat shock protein, mitochondrial-like protein	101	KPM11276
Heat shock protein 20-like protein 1	168	KPM02991
Heat shock protein 20-like protein 2	155	KPM04326
Heat Shock Protein 20-like protein 3	208	KPM04511
Heat Shock Protein 20-like protein 4	194	KPM09528
60 kDa Heat shock protein, mitochondrial-like protein	584	KPM06690
Sar s 28 (heat shock protein 70-like protein 1)	836	KPL97880
Sar s 28 (heat shock protein 70-like protein 2)	634	KPM03927
Sar s 28 (heat shock protein 70-like protein 3)	414	KPM07069
Sar s 28 (heat shock protein 70-like protein 4), partial	496	KPM07870
Sar s 28 (heat shock protein 70-like protein 5)	520	KPM08853
Sar s 28 (heat shock protein 70-like protein 6)	703	KPM10172
Sar s 28 (heat shock protein 70-like protein 7)	917	KPM10783
Sar s 28 (heat shock protein 70-like protein 8)	567	KPM11560
Heat shock protein 90-like protein 1	587	KPM03324
Heat shock protein 90-like protein 2	469	KPM07599
Heat shock protein 90-like protein 3	796	KPM09034

### Neurotransmitters and receptors

Incoming signals (chemo-, mechano-, photo-) travel along neurons as action potentials (APs). A membrane potential is maintained by the regulated pumping of sodium and potassium ions. Many sodium and potassium ion channel proteins have been predicted in the scabies mite proteome (Additional file 1: Table S7). Upon reaching the pre-synaptic membrane, the AP causes calcium channels to open and extracellular calcium diffuses into the neuron, which in turn triggers fusion of the synaptic vessel with the presynaptic membrane and the release of neurotransmitter that docks with the appropriate receptor on the postsynaptic membrane. There are four known classes of neural transmitters in insects: acetylcholine, biogenic amines, amino acids and peptides [39]. A gene for the receptor for the biogenic amine dopamine is present in the scabies mite genome (QR98_0022910). This neurotransmitter and GABA (γ- amino butyric acid) may play a role in salivary gland secretion in ticks [40, 41]. Could it be involved in scabies mite salivary secretion as well? We have identified a number of specific receptors for various neurotransmitters in these classes in the predicted *S. scabiei* proteome (Additional file 1: Table S8). These neurotransmitter receptor proteins include those predicted to bind acetylcholine, nicotinic, dopamine, serotonin, GABA and neuropeptide Y. Because the scabies mite proteome includes these neural receptors, presumably, these mites utilize the corresponding neurotransmitters.

Sequencing of the scabies mite genome predicted the presence of a neuropeptide receptor in the somatostatin/allatostatin family. In mammals, somatostatin is a peptide produced by neuroendrocrine neurons that has multiple functions including effecting neurotransmission [42]. Generally, it has inhibitory functions. Genes for the closely related neuropeptide allatostatins and their receptors have been found in the genome of the honeybee and in *D. melanogaster* and these share high homologies to somatostatin [43]. Allatostatins are thought to inhibit juvenile hormone synthesis in the honeybee brain. However, the presence of juvenile hormones in mites and ticks remains controversial [44]. Honeybee allatostatin [45] shares ~36 % identity with a hypothetical scabies mite protein (KPM07409). Thus, an allatostatin-like peptide hormone regulatory network probably also functions in *S. scabiei*, although its basic function remains unknown.

### Photoreception

Ability to detect environmental stimuli is important to survival of *S. scabiei*. Laboratory studies show that scabies mites respond positively to thermo-, chemo- and odor stimuli and will seek the source [46]. These mites respond to light even though they have no visible simple eyes on their body surface like some species of mites. However, simple light receptor cells could be located beneath the cuticle surface and stimulated when light passes through the clear cuticle. Opsin is a ubiquitous protein associated with light reception among most animals that respond to light. Opsin is coupled with the carotenoid light sensitive photopigment retinal to make the molecule rhodopsin [47]. Many predicted G-proteins, receptor-like proteins for rhodopsin and retinal, retinal degradation proteins, and molecules associated with the photochemistry of photoreception of electromagnetic radiation are present in the scabies mite proteome (Additional file 1: Table S9).

### Chemoreception

A classic host-seeking study has clearly shown that mites respond to host odor and are attracted to its source [46]. Several previous studies have also shown that scabies mites are attracted to 17 lipids that are present in or on the epidermis of mammalian skin [48]. Likewise, scabies mites are attracted by the odor from a host independent of body heat or contact with the skin and its various chemical components and physical properties [46]. Presumably these attractions facilitate location of a host and/or guide mites to specific areas of the host body.

In addition, our previous study revealed that scabies mites are attracted to several nitrogenous wastes and phenolic compounds that act in a pheromone-like manner and induce aggregation of these mites [49]. Ticks secrete nitrogenous wastes and phenolic compounds that act in a pheromone like-manner to promote assembly and other behavior [50]. The test methods used for the scabies mite behavior studies were not designed to determine the purpose of this assembly, but it may play a key role in attraction of sexes for mating. Early and ordinary scabies infestations involve few mites on the host so mite produced nitrogen wastes such as guanine, purine, adenine, allantoin, hypoxanthine, xanthine, uric acid, ammonium chloride, ammonium nitrate and ammonium sulfate and pheromones, likely play key roles in the reproduction of scabies mites during this time. The fact that scabies mites respond to these compounds indicates that they have chemoreceptors that recognize the volatile and contact compounds.

Taken together, all of these studies have shown that *S. scabiei* var. *canis* must have chemoreceptors. Like insects, it is expected that mites have odorant (OR), gustatory (GR) and ionotropic (IR) chemoreceptors. In the insects, and probably the Acari, chemoreceptors are usually located in hollow pegs or setae known as sensilla that are located on the antenna, terminal segments of the legs (tarsus), proboscis/mouthparts, wing margins, ovipositor and possibly pedipalps [51, 52]. Typically, these sensilla are perforated along the sides or at the tip and this allows stimulant molecules from the air (odor/pheromones) or contact (gustatory) to enter hemolymph in the sensilla bathing the neuron and bind to an odorant/tastant-binding protein that transports the stimulating molecule to the sensory receptor on the neuron [51]. The odorant/transporting complex or the released stimulant molecule activates the sensory receptor. Mites have hollow sensilla on the terminal segments of the legs that presumably function in a similar manner. It is not known if the oral cavity has chemoreceptors beneath the cuticle lining or if there are chemoreceptors on the pretarsal stocked empodia, chelicera, pedipalps and other body areas of scabies and other mites.

A scabies mite protein database search identified 13 predicted proteins that may be involved in chemoreception (Table 5). These include various sweet-taste receptor-like proteins, and a gustatory receptor-like protein. Our analysis of *S. scabiei* predicts fewer chemoreceptors in scabies mites than are reported in the genomes of some blood-sucking insects but approximately the same as for the body louse *Pediculus humanus corporis*. For example, the bedbug, *Cimex lectularius,* has genes coding for 49 olfactory receptors, 36 gustatory receptors and 30 ionotropic receptors [53]. The tsetse fly, *Glossina morsitans morsitans*, has 46 and 14 annotated gene loci for ORs and GRs, respectively [54]. The mosquitoes, *Anopheles gambiae* and *Aedes aegypti*, have many more ORs and GRs than both the tsetse flies and scabies mites. The genome for the human body louse contains ten odorant receptor genes [55].

**Table 5:** *Sarcoptes scabiei* var. *canis* proteins predicted to be involved in chemoreception. Proteins were identified by NBCI protein database search of “*Sarcoptes scabiei* [and] taste [or] sensory” on 1 Mar 16

Name assigned to *S. scabiei* predicted protein	# aa	Accession #
7 transmembrane sweet-taste receptor-like protein 1	140	KPM05630
7 transmembrane sweet-taste receptor-like protein 2	321	KPM06440
7 transmembrane sweet-taste receptor-like protein 3, partial	385	KPM09849
Class C metabotropic glutamate-like protein G-protein coupled receptor-like protein	187	KPM06441
Gamma-aminobutyric acid type B receptor subunit 1-like protein, partial	861	KPM06518
Glutamate receptor, metabotropic-like protein 2	164	KPM11384
Gustatory receptor-like protein	441	KPM04823
Metabotropic glutamate receptor 1-like protein 1, partial	429	KPM05105
Metabotropic glutamate receptor-like protein 3	526	KPM07738
Metabotropic glutamate receptor-like protein 4	476	KPM08699
Pheromone and odorant receptor-like protein	1650	KPM05712
Sensory neuron membrane protein 1-like protein	346	KPM11235
Sweet-taste receptor-like protein	429	KPM09147

Previous studies showed that scabies mites respond to host odor in the absence of carbon dioxide [46]. Whether or not CO_2_ by itself will arouse activity in scabies mites has not been determined. CO_2_ from a host is a stimulus for blood feeding mosquitoes and other insects [56, 57] and ticks [50]. Chemosensory neurons in *Drosophila* that are CO_2_-sensitive express the chemoreceptors Gr21a and Gr63a. The *An. gambiae* mosquito homologs are GPPRGR22 and GPRRGR24 [58]. The scabies mite predicted proteome does not have orthologs of the CO_2_ receptors from insects. Thus, if CO_2_ sensing occurs in scabies mites it utilizes an unknown pathway. However, scabies mites will seek a host in the absence of CO_2_. Genes for CO_2_ receptors have not been identified in the tick genome even though ticks do respond to CO_2_.

The family of ionotropic receptors can be located on post-synaptic membranes and form ion channels. These receptors have an extracellular domain that binds the neurotransmitter and other domains that cross the membrane and form the ion channel [59]. When activated by the neurotransmitter, the flow of ions through the pore is either increased or decreased. The scabies mite genome has 16 predicted “ionotropic receptor proteins” (Table 6). In contrast, bedbugs have 30 predicted ionotropic receptors [53], 24 of which have significant homology with 15 different *S. scabiei* var. *canis* proteins (Additional file 1: Table S10).

**Table 6:** *Sarcoptes scabiei* var. *canis* ionotropic receptors. Proteins were identified by NBCI protein database search of “*Sarcoptes scabiei* [and] ionotropic” on 24 Mar 16

Name assigned to *S. scabiei* predicted protein	# aa	Accession #
Glutamate receptor, ionotropic kainate 2 precursor-like protein 1	147	KPM04302
Glutamate receptor, ionotropic kainate 2 precursor-like protein 2	465	KPM04471
Glutamate receptor, ionotropic kainate 2-like protein 1	298	KPM02574
Glutamate receptor, ionotropic kainate 2-like protein 2	652	KPM04300
Glutamate receptor, ionotropic kainate 2-like protein 3	887	KPM05200
Glutamate receptor, ionotropic kainate 2-like protein 4	357	KPM05378
Glutamate receptor, ionotropic kainate 3-like protein 1	1102	KPL94547
Glutamate receptor, ionotropic kainate 3-like protein 2	883	KPM06129
Glutamate receptor, ionotropic kainate-like protein 1, partial	746	KPM03138
Glutamate receptor, ionotropic kainate-like protein 2	553	KPM05237
Glutamate receptor, ionotropic, kainate-like protein 3	180	KPM04472
Glutamate receptor, ionotropic, N-methyl D-aspartate-associated protein 1-like protein	223	KPM05160
Ionotropic kainate 2-like protein glutamate receptor-like protein	651	KPM04303
Kainate-selective ionotropic glutamate receptor-like protein 1, partial	461	KPL97052
Kainate-selective ionotropic glutamate receptor-like protein 2	234	KPM04473
Kainate-selective ionotropic glutamate receptor-like protein 3	1141	KPM09535

### Proteases and other enzymes

In a previous study, we used a variety of assays to characterize some of the enzymatic activities present in aqueous extracts of nine species of astigmatid mites [60]. Of the mite species tested, *S. scabiei* had the most limited enzymatic repertoire. Phosphatase, phosphohydrolase, esterase, aminopeptidase and glycosidase activities were detected in the scabies mite extract and proteins predicted to carry out these catalytic activities are all present in the proteome (Additional file 1: Table S11). Esterase lipase (C8) activity was detected in the extract while lipase (C14) activity was not even though the proteome is predicted to contain at least 20 lipase-like proteins of varying specificity.

Our previous study also failed to detect either chymotrypsin or trypsin catalytic activity in the scabies mite extract, a finding that was consistent with previous reports for other ectoparasites [61–63]. This is interesting since the *S. scabiei* var. *canis* proteome is predicted to contain one chymotrypsin-like protein and at least 13 trypsin-like serine proteases and ten serine protease-like proteins in addition to 18 serine protease-like proteins that are dust mite Group 3 allergen homologs (Sar s 3) (Additional file 1: Table S12).

**Fig. 1:**
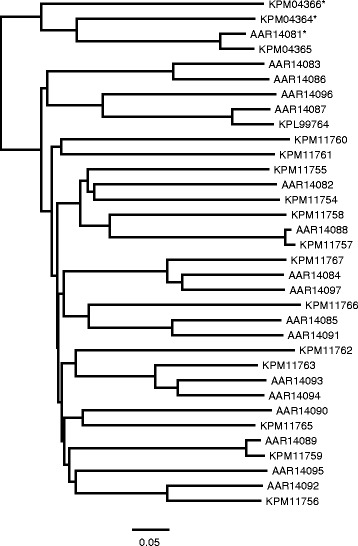
Neighbor joining tree of select serine proteases. Asterisks indicate members containing active site residues that are intact. Accessions beginning with “A” are *Sarcoptes scabiei* var. *hominis*, and those with “K” are *S. scabiei* var. *canis*. All putatively active members cluster together

**Fig. 2:**
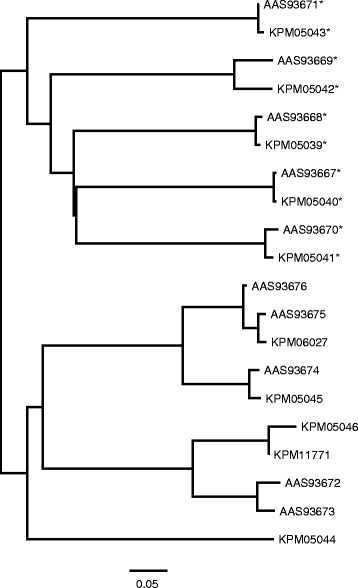
Neighbor joining tree of cysteine protease homologs. Asterisks indicate those containing active site residues that are intact. Accessions beginning with “A” are *Sarcoptes scabiei* var. *hominis*, and those with “K” are *S. scabiei* var. *canis*. All putatively active members cluster together

Scabies mites collected from human patients have previously been reported to possess a single, active Sar s 3 homolog and 16 structurally similar proteins (designated as Scabies Mite Inactivated Protease Paralogs or SMIPP-S) in which the catalytic triad is mutated so as to make them inactive [64]. The active protease is reported to be a digestive enzyme that is able to partially digest filaggrin, a protein found in human skin [65], while some of the inactive paralogs can inhibit the human complement system, presumably playing a role in allowing the parasite to evade the host’s immune defense systems [16]. Examination of the structures predicted for the various serine proteases of *S. scabiei* var. *canis* revealed that only five of 13 trypsin-like serine proteases, five of 10 serine protease-like proteins and two of the 18 Sar s 3 serine protease-like proteins had intact catalytic triads while the other members of these three groups of proteins had mutations similar to those in the SMIPP-S proteins (Additional file 1: Table S13). Phylogenetic comparisons of the Sar s 3 homologs from both human and dog scabies mites indicated that the sequences in both lineages that contain an active catalytic triad are derived from a common ancestral gene (Fig. 1). The remaining inactive protease genes appear to have undergone duplications and acquired inactivating mutations both before and after the split between the two mite lineages. Additionally, most of the mutant *S. scabiei* var. *canis* proteins did have intact substrate-binding sites. In the case of the ten predicted Sar s 1 cysteine protease-like proteins, five (KPM05039 – KPM05043) had intact catalytic triads and were orthologs of previously-reported Sar s 1 proteins (AAS93667–AAS93671) [66], suggesting an ancient and essential function for these five proteins (Fig. 2). The other five Sar s 1 family members had mutations in the catalytic triad like those in the SMIPP-C protein group, but the relationships of the *S. scabiei* var. *canis* homologs to those found in human scabies mites suggest that both recent and ancient duplications have occurred in the two scabies mite lineages.

The strain of scabies mites used to prepare the draft genome has been shown to be resistant to the acaricide permethrin [67, 68]. High levels of various detoxifying enzymes can be responsible for pesticide resistance in arthropods and we previously demonstrated that these mites had higher levels of esterase, cytochrome P450 monooxygenase and glutathione S-transferase (GST) activities than were present in permethrin-sensitive *S. scabiei* mites [68]. In addition to the esterases described above, these mites also are likely capable of producing 26 cytochrome P450-like proteins (Additional file 1: Table S2) and a variety of GSTs (Table 7).

**Table 7:** *Sarcoptes scabiei* var. *canis* glutathione S-transferases (GSTs). Proteins were identified by NBCI protein database search of “*Sarcoptes scabiei* [and] glutathione” on 22 Apr 16

Name assigned to *S. scabiei* predicted protein	# aa	Accession #
Glutathione S transferase-like protein 1	227	KPM02265
Glutathione S transferase-like protein 2	265	KPM02492
Glutathione S transferase-like protein 3	210	KPM03123
Glutathione S transferase-like protein 4	236	KPM03124
Glutathione S transferase-like protein 5	383	KPM11027
Glutathione S-transferase delta class 3	221	KPM09608
Glutathione S-transferase-like protein	227	KPM02264
Sar s 8 allergen (Glutathione S transferase mu-like protein 1)	262	KPM02280
Sar s 8 allergen (Glutathione S transferase mu-like protein 2)	200	KPM11584
Sar s 8 allergen (Glutathione S transferase mu-like protein 3)	219	KPM11585
Sar s 8 allergen (Glutathione S transferase mu-like protein 4)	219	KPM11586
Sar s 8 allergen (Glutathione S transferase mu-like protein 5)	219	KPM11587

In addition to the various enzymatic activities described above, scabies mites also appear to be able to synthesize the enzymes required to catalyze the vast spectrum of anabolic and catabolic reactions that are required by multicellular aerobic organisms (Additional file 2).

### Peritrophic membrane in the midgut

The peritrophic membrane in the insect and mite midgut protects the mucosa and may play a role in digestion. It contains a chitin and peritrophin protein complex. This membrane is sloughed as undigested food passes out of the midgut and it forms a membrane around (encapsulates) the fecal pellets. The fecal pellets can be seen in skin scrapes from scabietic patients and are an important observation for the diagnosis of this disease. We found at least 30 peritrophin homologs including a variety of proteins that contain the chitin-binding peritrophin-A domain (Table 8). One protein containing this chitin-binding domain is a homolog of the Group 23 dust mite allergens (Sar s 23). These proteins seem to provide evidence that even though *S. scabiei* may consume primarily a liquid diet of serum and digested epidermal stratum corneum, the peritrophic membrane (along with many digestive enzymes) has an important function in nutrient procurement, digestion and defecation in these mites.

**Table 8:** *Sarcoptes scabiei* var. *canis* proteins containing a peritrophin domain. Proteins were identified by NBCI protein database search of “*Sarcoptes scabiei* [and] peritrophin” on 20 Apr 16

Name assigned to *S. scabiei* predicted protein	# aa	Accession #
Chitin binding Peritrophin-A domain containing protein 1	101	KPM02161
Chitin binding Peritrophin-A domain containing protein 2	387	KPM03068
Chitin binding Peritrophin-A domain containing protein 3, partial	1167	KPM03793
Chitin binding Peritrophin-A domain containing protein 4, partial	379	KPM06347
Chitin binding Peritrophin-A domain containing protein 5, partial	452	KPM07124
Chitin binding Peritrophin-A domain containing protein 6	346	KPM07304
Chitin binding Peritrophin-A domain containing protein 7	222	KPM07429
Chitin binding Peritrophin-A domain containing protein 8	174	KPM07489
Chitin binding Peritrophin-A domain containing protein 9	309	KPM08965
Chitin binding Peritrophin-A domain containing protein 10, partial	583	KPM09937
Chitin binding Peritrophin-A domain containing protein 11	93	KPM11506
Chitin binding peritrophin-A-like protein 2	149	KPM09480
Chitin deacetylase-like protein 3	514	KPM11442
Chitinase-like protein 1, partial	1236	KPM05982
Chitinase-like protein 4	259	KPM08718
Chitinase-like protein 10, partial	653	KPM11497
Hypothetical protein QR98_0026710	691	KPM04228
Hypothetical protein QR98_0028980	485	KPM04450
Hypothetical protein QR98_0029710	290	KPM04522
Hypothetical protein QR98_0036850	289	KPM05225
Hypothetical protein QR98_0037360	256	KPM05275
Hypothetical protein QR98_0041930	348	KPM05724
Hypothetical protein QR98_0049080, partial	700	KPM06433
Hypothetical protein QR98_0065480	368	KPM08035
Hypothetical protein QR98_0075900	185	KPM09060
Hypothetical protein QR98_0086540	318	KPM10104
Peritrophin	486	AEA34990
Peritrophin-like protein	186	KPM09194
Sar s 15 allergen (chitinase-like protein)	571	KPM07813
Sar s 23 allergen (chitin binding domain containing protein)	92	KPM09573
Vesicle coat complex COPII, subunit SFB3-like protein	230	KPM03439

### Aquaporins/water balance and transport

Aquaporins are a family of transmembrane proteins that form pores through which water can flow in and out of cells and they are found in many different tissues and are in most organisms [69–73]. Aquaporins are essential components of the excretory system in Malpighian tubules of insects where they regulate water and solute concentrations [69, 73]. The scabies mite genome encodes two complete aquaporin genes, and fragments for three others were identified in the assembly. One of the complete genes encodes a traditional aquaporin (QR98_0011640 = KPM02745), while the second encodes an aquaglyceroporin (QR98_0057420 = KPM07253). It is likely that these proteins regulate both water balance and solute movement in this mite, concentrating nitrogenous waste in the Malpighian tubules and processing it for elimination.

### Excretion of nitrogenous wastes

Nitrogenous waste products are produced from the metabolism of proteins and purines. The nitrogenous waste produced by an organism is generally related to its solubility and the availability of water. In terrestrial ticks and mites, nitrogenous waste is shunted through the purine metabolism pathway, where guanine and xanthine are major waste products [74–76]. In *S. scabiei* var. *canis*, the gene for guanosine monophosphate synthase is present, which would allow the conversion of xanthosine monophosphate into guanosine monophosphate. Genes are also present for the multistep conversion of these monophosphates into their respective nucleobases through a purine nucleoside intermediate, or via the single step conversion to a nucleobase using phosphoribosyltransferases (Table 9). However, genes for guanine deaminase, which converts guanine to xanthine, were not found. Additionally, genes encoding xanthine dehydrogenase/oxidase were also not found. Thus, *S. scabiei* probably lacks the ability to produce urate. The pathway in *Ixodes* ticks (based on genomic information) is the same, thus the presence of guanine and xanthine in excrement likely results from the separate metabolism of the respective monophosphates, rather than the conversion of guanine to xanthine.

**Table 9:** *Sarcoptes scabiei* var. *canis* genes and proteins predicted to be involved in nitrogenous waste production

Gene locus	Predicted protein function	Accession #
QR98_0098200	GMP synthase	KPM11250
QR98_0088290	5′ nucleotidase	KPM10277
QR98_0037400	5′ nucleotidase	KPM05279
QR98_0082120	5′ nucleotidase	KPM09671
QR98_0000820	5′ nucleotidase	KPL94019
QR98_0039780	5′ nucleotidase	KPM05513
QR98_0057060	Purine nucleoside phosphorylase	KPM07218
QR98_0020510	Phosphoribosyltransferase	KPM03618
QR98_0100210	Hypoxanthine-guanine phosphoribosyltransferase	KPM11451

## Conclusions

Most biological processes in the scabies mite are not understood. Here, we have mined the draft *S. scabiei* var. *canis* genome and associated proteome in an attempt to identify homologs of genes and proteins already recognized to have known functions in some physiological processes in other animals. We have restricted this search to those genes and predicted proteins that are associated with basic biological processes or requirements. We were particularly interested in genes that encode for proteins known to be associated with vital biological processes such as immune modulation, obtaining oxygen for aerobic respiration and oxidative metabolism, sensory reception and locating a host, neuronal transmission, stressors (heat shock proteins), molting, movement, excretion and water balance, nutrient procurement and digestion. We have not attempted to identify gene groups coding for enzymes and proteins associated with the many anabolic and catabolic processes and biochemical pathways such as 2nd messenger signaling, regulation of gene expression, transcription, translation, Golgi and mitochondrial function, energy metabolism, endocrine system function and hormone synthesis, secretion, etc.

This research is obviously limited by the data that are already available for other organisms in the public databases. At this time there are ~3350 *S. scabiei* var. *canis* genes without orthologs in other mite species and ~4100 predicted proteins in scabies mites identified as “hypothetical” since they have no matches in any database. This does not mean that all these genes and proteins are unique to scabies mites although some probably are. As more genomes for other animals and in particular arthropods are sequenced and the data made available, the list of unassigned genes and proteins will likely shrink. For now, we are able to use the available data to speculate on some biological processes for scabies mites that may help understand their biology.

## Additional files

Additional file 1: Tables S1-S13. Complete results of the various database searches described in the text. (XLSX 101 kb) Additional file 2: BLAST2GO results generated on 25 Feb 2016 as described in the Methods. Data are sorted alphabetically by GO Biological Process. (XLSX 192 kb)
